# A shift in species niche through autopolyploidy: from sexual to vegetative reproduction in *Saxifraga oppositifolia*

**DOI:** 10.1093/aob/mcag116

**Published:** 2026-04-29

**Authors:** Ingrid Vesterdal Tjessem, Anne Krag Brysting, Trond Reitan, Simen Salomonsen Hjelle, Pernille Bronken Eidesen

**Affiliations:** Department of Biosciences, University of Oslo, PO Box 1066, Blindern, Oslo 0316, Norway; Department of Arctic Biology, The University Centre in Svalbard, PO Box 156, Longyearbyen 9171, Norway; Natural History Museum, University of Oslo, PO Box 1172, Blindern, Oslo 0318, Norway; Department of Biosciences, University of Oslo, PO Box 1066, Blindern, Oslo 0316, Norway; Department of Biosciences, University of Oslo, PO Box 1066, Blindern, Oslo 0316, Norway; Department of Geosciences, University of Oslo, PO Box 1028, Blindern, Oslo 0316, Norway; Department of Arctic Biology, The University Centre in Svalbard, PO Box 156, Longyearbyen 9171, Norway; Department of Arctic and Marine Biology, The Arctic University of Norway, PO Box 6050, Langnes, Tromsø 9037, Norway; Department of Biosciences, University of Oslo, PO Box 1066, Blindern, Oslo 0316, Norway; Department of Arctic Biology, The University Centre in Svalbard, PO Box 156, Longyearbyen 9171, Norway

**Keywords:** Autopolyploidy, cytotype, habitat segregation, niche shift, phenotypic variation, polyploid speciation, reproductive strategy, reproductive trait, *Saxifraga oppositifolia*, sexual reproduction, vegetative propagation

## Abstract

**Background and Aims:**

Polyploidy has been essential in the evolution of angiosperms, facilitating novel adaptations, enhancing speciation and contributing to the establishment of larger evolutionary lineages. However, newly formed polyploids require sufficient niche differentiation to survive and coexist with the diploid ancestor. Polyploids are commonly reproductively challenged, which may lead to rapid development of asexual reproduction, which may, in turn, lead to reproductive barriers or ecological niche divergence. Through this study, we investigate whether autopolyploidization has induced a shift from sexual reproduction to vegetative propagation in the arctic–alpine *Saxifraga oppositifolia* (Saxifragaceae) and thus facilitated the establishment of polyploids in mixed-cytotype populations.

**Methods:**

We sampled data on capsule and seed production, seed germination, rooting ability, and leaf production among cytotypes through a combination of growth chamber experiments and *in situ* observations in a field set-up across different habitats (ridges, slopes, riverbeds) in Svalbard including >700 georeferenced *S. oppositifolia* plants with known cytotype (diploid, triploid or tetraploid). Through hypothesis testing and an explorative model search for relevant covariates (e.g. growth form, habitat, soil type), we tested the effect of cytotype on sexual and vegetative reproductive outcomes.

**Key Results:**

Polyploids had lower sexual reproductive investment (i.e. capsule production and seed production) and higher vegetative investment (i.e. leaf production and rooting ability), showing that polyploidy has significantly affected both sexual reproduction and vegetative propagation.

**Conclusions:**

Our findings confirm a shift in reproductive strategy towards less sexual investment and enhanced vegetative investment in triploid and tetraploid individuals. This shift may enhance polyploid survival in habitats with shorter growing season and limited pollinator availability, and thus explains their higher frequency in such habitats. These results support autopolyploidy as an evolutionary mechanism that may allow Arctic species to adapt to ongoing, rapid changes.

## INTRODUCTION

Polyploidy has played an important role in angiosperm evolution by facilitating new adaptations and thus promoting speciation and new evolutionary lineages (Jiao *et al*., 2011; Husband *et al*., 2013; Chanderbali *et al*., 2016; Soltis and Soltis, 2016; Van de Peer *et al*., 2017; Heslop-Harrison *et al*., 2023). The formation of polyploid individuals is rather common, particularly under stressful conditions that may increase the risk of meiotic errors and production of unreduced gametes (Mable, 2004; Van de Peer *et al*., 2021). However, their contribution to evolution further requires the establishment of a population, reproduction and heredity. The path leading to the evolutionary success of polyploids comes with high costs and has been referred to as a paradox seen in light of modern coexistence theory (Mortier *et al*., 2024). Newly formed polyploids (neopolyploids) are often reproductively challenged through chromosomal pairing problems (Grant, 1981; Ramsey and Schemske, 2002) or endosperm incompatibility (Köhler *et al*., 2010). Furthermore, neopolyploids must overcome competition from their diploid progenitors. Thus, successful establishment of neopolyploids is often associated with shifts in reproductive strategy towards increased self-compatibility, apomixis or vegetative propagation (Herben *et al*., 2017; Van Drunen and Husband, 2018*a*; Wakui and Kudo, 2021; Van Drunen and Friedman, 2022), and ecological niche divergence (Husband, 2000; Karunarathne *et al*., 2018). To better understand polyploid establishment and the interplay among abiotic and biotic factors leading to stable coexistence with progenitors, we need good model systems and a holistic research approach (Segraves and Anneberg, 2024).

Polyploidy may originate within the same species (autopolyploidy) or from the hybridization of two different species (allopolyploidy). The evolutionary impact of autopolyploidy versus allopolyploidy has long been debated (Müntzing, 1936; Barker *et al*., 2016; Doyle and Sherman-Broyles, 2017; Kauai *et al*., 2023), but at present there is no doubt that both are important evolutionary mechanisms (Konečná *et al*., 2021). However, autopolyploids are easily overlooked as they usually lack morphological differences relative to their diploid progenitors (Soltis *et al*., 2007, 2014; Parisod *et al*., 2010). Although the body of research literature regarding the ecology and evolution of polyploids has strongly expanded over the last few decades (Segraves and Anneberg, 2024), our knowledge regarding autopolyploidy is less developed (Spoelhof *et al*., 2017; Lv *et al*., 2024), and natural model systems for empirical studies related to autopolyploidy are particularly scarce (Konečná *et al*., 2021).

*Saxifraga oppositifolia* L. (Saxifragaceae) is a common, arctic–alpine plant species, and is widely regarded as an important model system for the evolution of arctic–alpine plants (Holderegger and Abbott, 2003). Diploids are registered throughout the distribution range except in Beringia, where only tetraploids are documented (Elven *et al*., 2023). Besides diploids, triploids and tetraploids are also registered in Canada, Greenland, and the high Arctic Archipelago Svalbard (Eidesen *et al*., 2013, 2025; Elven *et al*., 2023; Gago and Clemente, 2023). Investigations from Svalbard have revealed that mixed-ploidy populations, including diploids, triploids and tetraploids, are common (Müller *et al*., 2012; Eidesen *et al*., 2013, 2025).

**Fig. 1.**
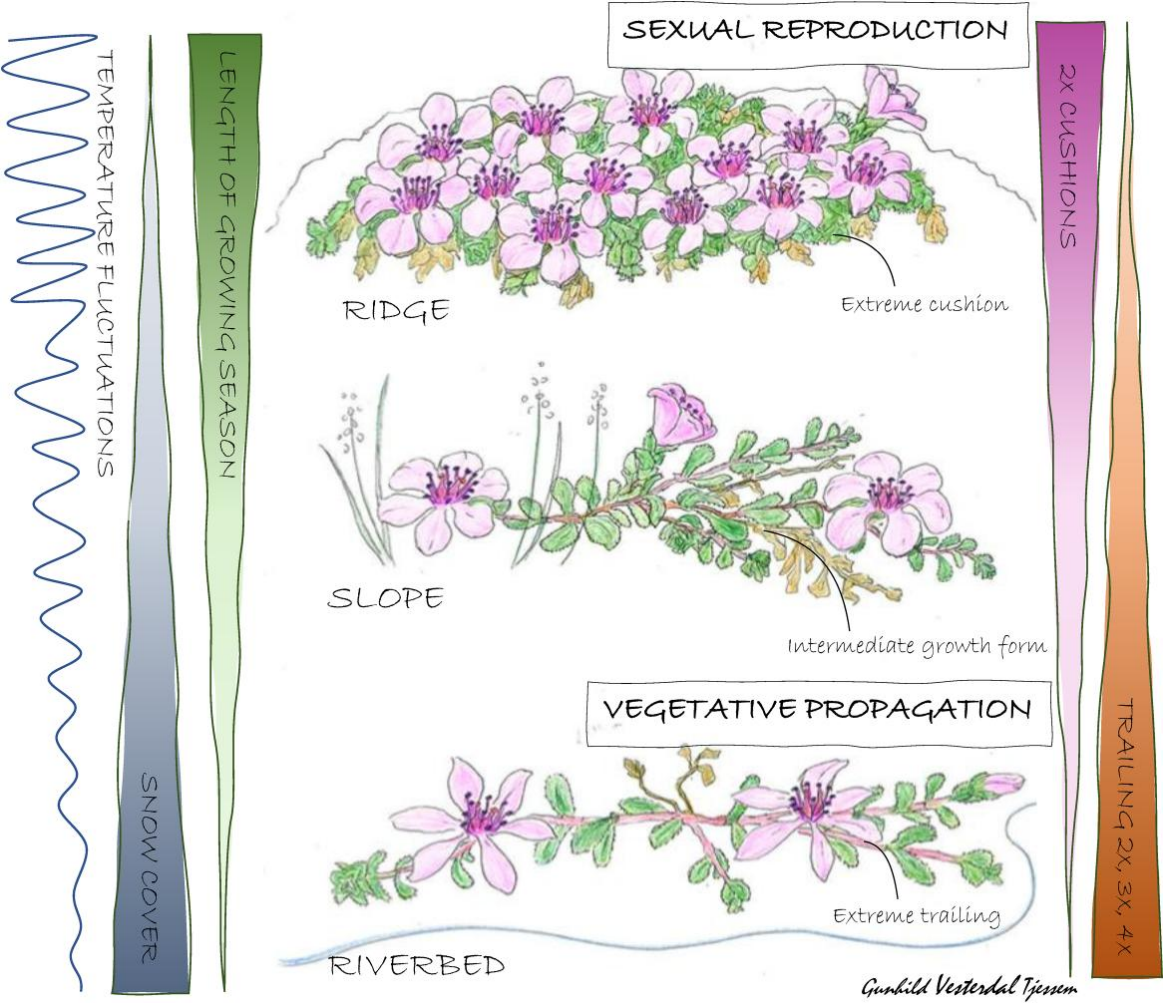
Phenotypic variation in the Arctic–Alpine *Saxifraga oppositifolia*. Only diploids form dense cushions, whereas all cytotypes form trailing plants (diploids – 2*x*, tetraploids – 4*x*, and triploids – 3*x*). Cytotype frequency differs among habitat types; diploids occur in all habitats, whereas polyploids are rarely found on ridges. Consequently, cushions are more common on exposed ridges, whereas trailing plants are more common in sheltered riverbeds. Ridges have an earlier snowmelt and a longer growing season, compared to riverbeds with a longer snow cover and shorter growing season. Slopes have a higher vegetation cover compared to ridges and riverbeds, and with higher biomass production, the soil typically contains higher levels of organic matter. Ridges are more weather-exposed, with more fluctuating temperatures compared to more sheltered riverbeds. Adapted from an original design by Ingrid Vesterdal Tjessem; drawing by Gunhild Vesterdal Tjessem. ; 2026 by Ingrid Vesterdal Tjessem and Gunhild Vesterdal Tjessem.

A large variation in growth form and habitat is found in *S. oppositifolia*. Growth form varies from fully trailing individuals to compact cushions (Fig. 1). The range of ecological variation spans a variety of habitats from dry early-melting sites to wet late-melting sites. Former studies on *S. oppositifolia* from Svalbard and the Canadian Arctic have associated variation in growth form and reproductive and physiological traits with habitat variation (Teeri, 1973; Crawford and Abbott, 1994; Crawford *et al*., 1995; Kume *et al*., 1999). The trailing growth form has, for instance, been associated with snow-protected, moist habitats, whereas the cushion growth form has been associated with dry, wind-exposed ridges (Crawford *et al*., 1995).

The existence of triploids and tetraploids has been known for almost a century (Elven *et al*., 2023), yet, until recently, the few attempts to combine cytotype information with morphological or ecological variation revealed no clear associations (Brysting *et al*., 1996; Aiken *et al*., 2005). Later studies from Svalbard have, however, shown that significant genetic and phenotypic variation in *S. oppositifolia* is related to autopolyploidy, that different growth forms are strongly associated with cytotype and that cytotype frequencies differ among habitats (Müller *et al*., 2012; Eidesen *et al*., 2013, 2025).

Diploids exhibit all growth forms across all habitats, whereas triploids and tetraploids express only trailing growth forms and have a narrower habitat range (Eidesen *et al*., 2013, 2025) (Fig. 1). Although some plasticity in growth form may occur, current knowledge suggests that growth form is mainly determined by genetics (Crawford *et al*., 1995; Eidesen *et al*., 2013, 2025). Diploids totally dominate on exposed ridges and unstable scree slopes, whereas triploids and tetraploids are found in less exposed habitats and dominate or are found in equal frequencies as diploids in mixed populations situated in sloping heath vegetation or stabilized riverbeds (Eidesen *et al*., 2025). Soil parameters (such as pH and grain size) correlate with vegetation composition associated with these different habitats (Eidesen *et al*., 2025), suggesting edaphic preferences may influence establishment and frequency distributions at larger spatial resolution.

Reproductive outcome in terms of flowers has been shown to differ between cytotypes. Although the probability of a flower turning into a capsule was found to be the same within the same year, regardless of cytotype, Eidesen *et al*. (2025) concluded that diploids flower more frequently and produce more flowers than polyploids, regardless of growth form or habitat. Thus, if polyploids invest less in flowers, they should invest more in something else, which has not yet been studied. A study investigating vegetative propagation in *S. oppositifolia* found that cuttings from trailing plants rooted better than those from cushion-forming plants (Kume *et al*., 1999; Hagen, 2002). However, neither of these studies accounted for different cytotypes, and all cytotypes form trailing plants (Eidesen *et al*., 2025).

In this study, we investigate whether cytotypes of *S. oppositifolia* differ in reproductive strategy and how this may be related to niche differentiation among diploids and polyploids. We used a pre-established field set-up across different habitats in Svalbard, with >700 georeferenced marked plants with known cytotype. Through *in situ* observation and collection, and growth experiments in climate chambers, we obtained data on capsule and seed production, seed germination, rooting ability and leaf production. We investigated whether cytotype can explain variation in reproductive strategy (i.e. sexual vs vegetative), beyond the variation explained by phenotypic variation (e.g. growth form), site characteristics (e.g. habitat, soil type) or other potential covariates. Based on our results, we discuss whether autopolyploidization has induced a shift in reproductive strategy, from sexual reproduction to vegetative propagation, and thus facilitated the establishment of polyploids and niche differentiation in *S. oppositifolia*.

## MATERIAL AND METHODS

### Study area

**Fig. 2.**
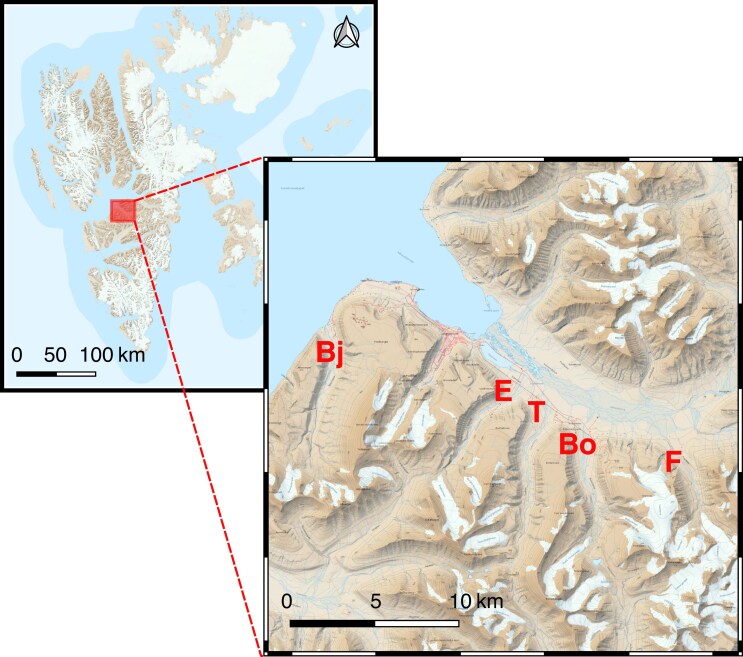
Map of the Archipelago of Svalbard (74–81°N, 10–35°E) with the study area (inset), including the five valleys in which fieldwork was conducted. Bj, Bjørndalen; E, Endalen; T, Todalen; Bo, Bolterdalen; F, Foxdalen.

From June to August 2020, fieldwork was conducted in the vicinity of Longyearbyen, innermost Adventfjorden, in the western part of Spitsbergen, the largest of the Arctic islands in the Archipelago of Svalbard, Norway (Fig. 2). Based on vegetation composition and summer temperatures, the area is included in the Arctic bioclimatic subzone C, and commonly characterized by open patchy vegetation with 5–50 % cover of vascular plants (Walker *et al*., 2005). Data were collected from a study system established in 2018, referred to as the SO-field study (for a detailed description of the SO-field study, see Eidesen *et al*., 2025). The study system included transects in five successive valleys representing a gradient from coast to inland (Fig. 2). The transects each included three plots representing a gradient of snow distribution (ridge/scree slope, sloping heath and stabilized riverbed). Each plot contained 48 tagged *S. oppositifolia* plants, for which cytotype had already been determined by flow cytometry analyses (Eidesen *et al*., 2025).

### Data collection

#### Sexual reproduction – capsule and seed production

We obtained data on capsule and seed production for all 723 tagged *S. oppositifolia* plants. Capsule production (i.e. capsule:plant ratio) was estimated based on *in situ* capsule counts in August 2020. Up to ten capsules were collected from each plant (an upper limit of ten capsules was set to minimize the negative impact on the plants). Only closed capsules were collected to ensure that seeds had not already been dispersed. The capsules were stored in paper bags in a cold room (4 °C) until they were dissected, and the seeds were counted by hand to obtain a measure of seed production (i.e. seed:capsule ratio).

#### Sexual reproduction – seed germination

Initially, only plants with at least 25 collected seeds were included in the seed germination experiment (51 diploid and four tetraploid plants). To improve the representation of tetraploids, two additional plants with a lower seed count than the initial seed limit were included (with 22 and 16 seeds, respectively). Triploids were not represented in the experiment due to low sexual reproductive outcome (<1 seed per capsule on average). The total seed weight from each plant (i.e. the 25 seeds) was measured before the seeds were surface-sterilized by treating them with 70 % ethanol, bleach (20 % chlorine, 0.1 % Tween20) and wash solution (0.001 % Tween20) for 5 min at each step (Lindsey *et al*., 2017). A 0.1 % agar solution was added to the seeds before they were transferred to Petri dishes with 0.5 MS growth medium including 2 % sucrose (Murashige and Skoog, 1962). After a 3-week stratification period with 4 °C and dark, the seeds were placed in conditions with 24 h of light and 16 °C for the first week, 18 °C for the second week and 20 °C for the rest of the period. During the second week, 500 µL double distilled H_2_O was added to each plate under sterile conditions to prevent the seedlings from drying out. After 7 weeks, the germinated seedlings were counted, and seed germination (i.e. germination percentage) was estimated.

#### Vegetative propagation – rooting ability and leaf production

To evaluate vegetative propagation, the rooting ability of cuttings and subsequent leaf production were compared among cytotypes through a potting experiment. Although rooting ability was considered to be a more direct measure of vegetative propagation, leaf production was also included since it may reflect vegetative resource allocation. Cuttings were collected *in situ* from tagged plants in the SO-field study. Two cuttings were taken from each of 168 plants, resulting in 336 cuttings that represented 56 diploids, 50 triploids and 62 tetraploids across the three habitat types (ridge, slope, riverbed). The only criterion for collecting cuttings was that a plant had a minimum of two branches that each measured at least 10 cm. The collected cuttings were stored in a refrigerator until the experiment started.

The experimental set-up included two different soil treatments: 168 cuttings in soil A – a more alkaline and mineral-rich soil, and 168 cuttings in soil B – a more acidic and organic soil. Both soil mixes were made by Cornell’s ratio with two parts peat moss and one part perlite (by volume) (Boodley and Sheldrake, 1972). The peat moss contained 22.5 % sieved sand and 8 % sieved organic soil for soil A and B, respectively. The pots were watered with 200 mL water and then well drained. Cuttings were shortened with a sterilized scalpel to make a final length of 5 cm and placed into separate pots: for each plant, one cutting was placed in mineral soil (A) and the other in organic soil (B).

The cuttings were given 100 mL additional water and placed in a climate chamber at 5–6 °C with 24 h of daylight. One week later, they were moved to a 14–15 °C climate chamber with the same light conditions. The cuttings were watered with ten sprays from a spray bottle every second day. After 9 weeks, the presence of rooting in the 265 surviving cuttings was scored. Five rooting categories (rooting stages 0–4), ranging from no visible roots to clearly rooting (Table 1), were defined based on the observed variation in rooting ability after the experiment was conducted. Additionally, leaf production was recorded as a binary trait (presence/absence).

**Table 1.** Five categories (rooting stage 0–4) were used in the rooting ability experiment to classify *Saxifraga oppositifolia* cuttings and evaluate their potential for vegetative propagation.

0	1	2	3	4
No visible roots.	Rooting, but only visible in a stereo microscope.	Some indication of new roots.	Rooting, but the new root/roots are small.	Clearly rooting. Roots are branching.
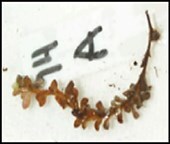	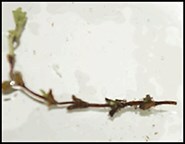	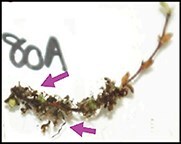	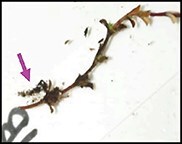	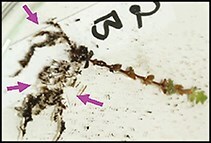

The images include cuttings scored into rooting stage according to the varying degree of documented roots marked by arrows.

### Statistical analyses

The objective of this study was to test whether cytotype had an effect on reproductive strategy. The five reproductive outcomes we analysed were (A) capsule production, (B) seed production, (C) seed germination, (D) leaf production and (E) rooting ability (Table 2). These five reproductive outcomes served as response variables in five regression analyses. The nature of the outcomes (unlimited count outcomes, limited count outcomes, binary outcomes or ordinal categories) determined the type of regression performed (see Table 2). However, to prevent misattributing the effects of cytotype to other correlated variables, we first performed an explorative analysis with other potentially relevant covariates (see Table 3 and Supplementary Data Table S1, Appendix S1 for the covariates included in each analysis). From the available sampled data, both from the *in situ* observation and collection, and growth experiments in climate chambers, potentially relevant covariates were chosen according to ecological considerations and their presumed ability to affect each reproductive outcome.

**Table 2.** Measures on sexual and vegetative reproductive strategy obtained from *Saxifraga oppositifolia* and used in five explorative analyses (A–E), together with their respective response variable, range and regression type.

Analysis		Response variable	Outcome range	Regression
A	Capsule production (sexual reproduction)	Number (integer) of capsules per plant	1–204 capsules per plant	Poisson
B	Seed production (sexual reproduction)	Number (integer) of seeds per capsule	0–338 seeds per capsule	Poisson
C	Seed germination (sexual reproduction)	Germinated vs. not germinated out of total number of seeds per plate	0–14 germinated seeds out of 25 seeds per plate	Binomial (*k* out of 25)
D	Leaf production (vegetative propagation)	Presence vs. absence of new leaves	Presence or absence of new leaves	Binomial (binary)
E	Rooting ability (vegetative propagation)	Number (integer) of cuttings in ordered rooting ability stages	Five rooting ability stages (Table 1)	Ordinal

**Table 3.** Measures on sexual and vegetative reproductive strategy obtained from *Saxifraga oppositifolia* and used in five explorative analyses (A–E, Table 2), along with their potential relevant covariates and the total number of single and interacting covariates, which presumably could explain variation in the five reproductive outcomes of *Saxifraga oppositifolia* (Table 2).

Analysis		Covariates (single covariates)	Total number of covariates (single and interacting)
A	Capsule production (sexual reproduction)	Growth form, Habitat, ID, Plot, Transect	8
B	Seed production (sexual reproduction)	Growth form, Habitat, ID, Plot, Transect, No. of flowers	13
C	Seed germination (sexual reproduction)	Growth form, Habitat, ID, Plot, Transect, No. of seeds, Seed weight	19
D	Leaf production (vegetative propagation)	Habitat, Plot, Soil type	5
E	Rooting ability (vegetative propagation)	Habitat, Plot, Soil type	5

ID corresponds to unexplained variation (i.e. a per-sample random effect).

Though the effect of potentially relevant covariates can be of interest in themselves, we wanted to test whether cytotype explained some of the variation that had previously been assumed to depend solely on phenotypic variation, site characteristics or other covariates. Thus, in the context of testing the effect of cytotype on reproductive strategy, phenotypic variation (e.g. growth form), site characteristics (e.g. habitat, soil type) or other potential covariates can be labelled nuisance variables. By performing an explorative analysis on other potential covariates prior to a hypothesis test on cytotype, instead of performing hypothesis testing on all potential covariates, including cytotype, we control for other potential covariates to reduce the risk of false positives (Type I error) and false negatives (Type II error) (Smith and Ebrahim, 2002).

For analyses A–C, the response variables were numbers ranging up to more than 1 (rather than binary outcomes, which have no middle position, or ordinal categories), making overdispersion possible. In these analyses, we included a per-sample random effect as one of the potential covariates, as this is identical to overdispersion (see e.g. Harrison, 2015). Hereafter, we term overdispersion unexplained variation, as per-sample random effects are noise terms without discernible patterns.

The explorative analyses on other potential covariates were carried out by a model search for a set of potential covariates (Table 3 and Supplementary Data Table S1, Appendix S1), consisting of per-sample random effects (where applicable), the measured variables and interactions (two-way) between them. We used the second-order corrected Akaike Information Criterion (AICc) as the model selection criterion to account for small sample size (Hurvich and Tsai, 1989), and to minimize the prediction variance. We always kept the best model according to the lowest AICc. When the number of potential covariates was low (five or fewer, analyses D and E), we used an exhaustive search algorithm called *regress.ic.dredge* (for five potential covariates, we have 2^5^ = 32 possible models). For response variables with more than five potential covariates (analyses A–C), we used a stepwise search algorithm called *regress.ic.search* (see Data Availability for both algorithms). These search functions look through various generalized linear models (GLMs using the *lme4* package for analyses A–D) or cumulative link models (CLMs using the *ordinal* package for analysis E) for ordinal analysis that can include mixed effects.

To investigate model selection uncertainty, we retrieved the top five models in each explorative analysis. We calculated the corresponding AICc weight, or ‘weight of evidence’, which was interpreted as the probability for a model to be considered the best model and to consider the prediction uncertainties between models (Burnham and Anderson, 2004) (Supplementary Data Tables S2–S6, Appendix S2).

After the explorative analyses, we added cytotype to these full models to test the central hypothesis of cytotype as an explanatory variable using the chi-squared likelihood-ratio test with Bonferroni correction (Dunn, 1961). Cytotype was coded as a binary variable (diploids vs. polyploids) and as a three-level factor (diploid, triploid, tetraploid). Both variables were considered and hypothesis tested to see which best represented variation in cytotype. Cytotypes were included in the final model if the variable was significant with 95 % confidence (*P* < 0.05). We considered the final model from the exploratory analysis as the best model, including cytotype when it was statistically significant and excluding it when it was not. The final model coefficient estimates were evaluated along with their respective contribution to the model and the objectives. Variance contributions (%) were estimated as var(coefficient estimate * variable) for the fixed effects, while the variance contributions from random effects were obtained directly from the model summaries.

A goodness-of-fit test for CLMs from the R package *ordinal* (Christensen, 2023) was run to test if the assumption of proportional odds was held for the final model in analysis E. No significant *P*-values were found for the independent variables (i.e. no evidence was found of non-proportional odds).

All statistical analyses were performed using R v.4.5.2 (R Core Team, 2025), *lme4* (Bates *et al*., 2015) for analyses A–D and *ordinal* (Christensen, 2023) for analysis E.

## RESULTS

### Effect of cytotype on reproductive strategy

**Fig. 3.**
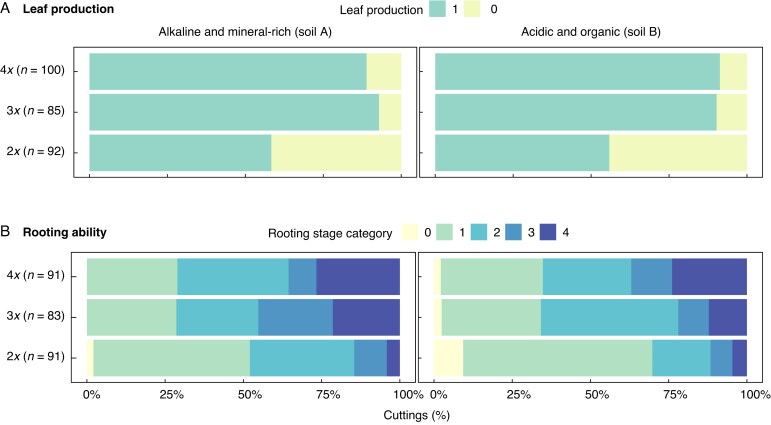
Percentage of diploid (2*x*), triploid (3*x*) and tetraploid (4*x*) *Saxifraga oppositifolia* cuttings that after 9 weeks were categorized based on (A) absence (0) or presence (1) of leaf production in the alkaline and mineral-rich soil (soil A) and the acidic and organic soil (soil B), and (B) rooting ability (for rooting stage categories 0–4, see Table 1) in soil A and soil B.

**Fig. 4.**
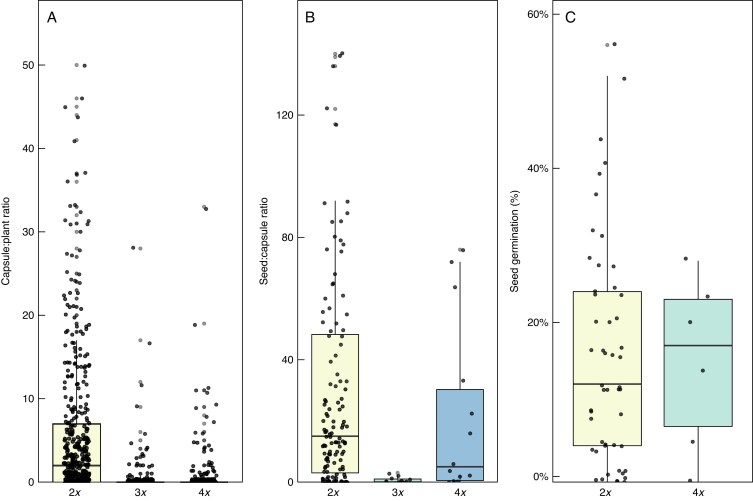
Variation in measures of sexual reproductive strategy in different *Saxifraga oppositifolia* cytotypes (2*x*, 3*x*, 4*x*). (A) Capsule production. (B) Seed production. (C) Seed germination. Boxplot includes median (horizontal line), interquartile range (IQR) (box) and range (vertical line).

Cytotype had a significant effect (*P* < 0.05) on all the investigated outcomes on sexual reproduction, except seed germination (Table 4, analysis C), and both investigated outcomes on vegetative propagation (Table 4). Polyploids invested more in vegetative propagation, with more than five (tetraploids) and three (triploids) times higher percentage points of cuttings in rooting stage 4 than diploids (Fig. 3B). Leaf production was higher in polyploids, with more than 10 and 20 percentage points compared with diploids in the alkaline and mineral-rich soil (A) and acidic and organic soil (B), respectively (Fig. 3A). By contrast, lower capsule and seed production in polyploids suggested that they invested less in sexual reproduction compared with diploids (Fig. 4A, B). Diploids had the highest average capsule production (capsule:plant ratio 2.5) (Fig. 4A) and seed production (seed:capsule ratio 15) (Fig. 4B). Triploids produced almost no capsules (Fig. 4A) or seeds (Fig. 4B), and thus were not represented with germination percentage (Fig. 4C). Tetraploids also had an average capsule production close to zero (Fig. 4B), but the few capsules produced provided an average seed production above zero (seed:capsule ratio 5) (Fig. 4B). Average seed germination was higher in tetraploids than in diploids; however, the data had a large spread and the range in both cytotypes overlapped (Fig. 4C).

**Table 4.** To test the effect of polyploidy on reproductive strategy in *Saxifraga oppositifolia*, the best model from the explorative analyses (A–E) was used as the null-hypothesis with an added ploidy regression coefficient as the alternative hypothesis.

Analysis		Polyploidy significance (*P* < 0.05)
A	Capsule production (sexual reproduction)	**Yes (*P* < 0.001)**
B	Seed production (sexual reproduction)	**Yes (*P* < 0.001)**
C	Seed germination (sexual reproduction)	No (*P* = 0.813)
D	Leaf production (vegetative propagation)	**Yes (*P* = 0.001)**
E	Rooting ability (vegetative propagation)	**Yes (*P* = 0.011)**

The table shows *P*-values from the likelihood-ratio chi-squared tests.

**Fig. 5.**
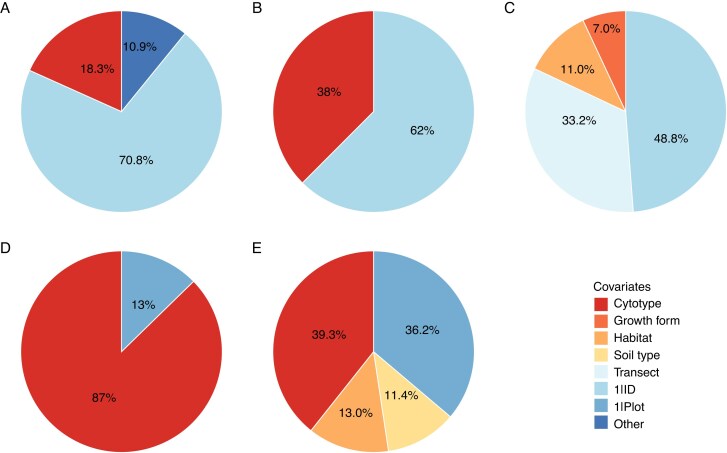
Variance contribution (%) from the covariates (fixed and random effects) in the best models of sexual and vegetative reproductive strategy in *Saxifraga oppositifolia*. (A) Generalized linear mixed model (GLMM) of capsule production (capsule:plant ratio). (B) Generalized linear model (GLM) of seed production (seed:capsule ratio). (C) GLMM of seed germination (germinated seeds:total seeds ratio). (D) GLMM of leaf production. (E) Cumulative link mixed model (CLMM) of rooting ability (Analysis E). The key shows all the tested covariates (fixed and random effects). ‘1|ID’ corresponds to unexplained variation (i.e. a per-sample random effect), and ‘Other’ represents interactions (two-way) between the listed covariates (i.e. correlated covariates with both varying slope and intercept).

**Fig. 6.**
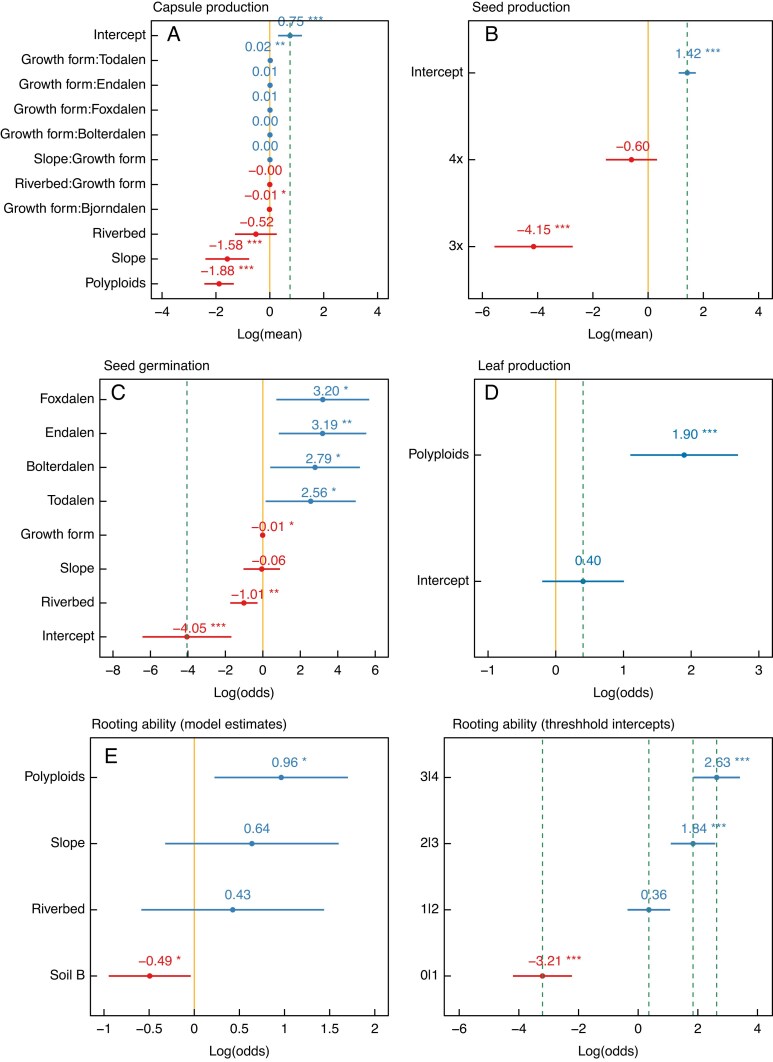
Model estimates (dots) and 95% confidence intervals (lines) for the fixed effects and interactions (two-way) that were included in the best models of sexual and vegetative reproductive strategy in *Saxifraga oppositifolia*. The yellow line marks zero effect on the *x*-axis and thus the boundary between a positive (blue) and a negative (red) effect. The dashed seagreen line marks the intercept estimate for each model. *P*-values are summarized as significance codes when *P* < 0–0.001 (***), *P* < 0.001–0.01 (**) or *P* < 0.01–0.05 (*). (A) GLMM of capsule:plant ratio. The intercept corresponds to diploids [diploids (2*x*) vs. polyploids (3*x* and 4*x*)], ridge (habitat) and Bjørndalen (transect). (B) GLM of seed:capsule ratio. The intercept corresponds to diploids (2*x*) [cytotype (2*x*, 3*x*, 4*x*)]. (C) GLMM of germination. The intercept corresponds to ridge (habitat) and Bjørndalen (transect). (D) GLMM of leaf production. The intercept corresponds to diploids [diploids (2*x*) vs. polyploids (3*x* and 4*x*)]. (E) CLMM of rooting ability. Estimates (left) give the change in unit at each threshold intercept (right) when going from intercept to a coefficient value. The CLMM threshold intercepts (right) summarize the reference value on the *y*-axis for diploids (diploids vs. polyploids), ridge (habitat) and soil A (soil type) between category 0 and 1 for threshold 0|1, and similar for 1|2, 2|3 and 3|4.

The best model for seed production included three covariates: cytotype, unexplained variation, and an interaction between growth form (random slope) and plot (Supplementary Data Table S7, Appendix S2). Given the complexity of partitioning variance for an interaction involving a random slope, we excluded this term from the variance decomposition presented in Fig. 5B. Cytotype was the only fixed effect included in the model and explained 38 % of the variation in seed production (Fig. 5B). Triploids had the lowest seed production, with a negative model estimate (*b* = −4.15, s.e. = 0.72) relative to the positive model estimate for diploids (intercept: 1.42, s.e. = 0.16) (Fig. 6B). The 95 % confidence interval for tetraploid seed production overlapped zero (*b* = −0.60, s.e. = 0.47), and the difference relative to diploids was not statistically significant (*P* > 0.05) (Fig. 6B). Leaf production (Fig. 5D) was almost entirely (87 %) explained by variation in cytotype. Cytotype further explained 39.3 % of the variation in rooting ability (Fig. 5E) and 18.3 % of the variation in capsule production (Fig. 5A).

### Effect of phenotypic variation and site characteristics on reproductive strategy

The two different soil treatments resulted in a similar distribution of leaf production between cytotypes (Fig. 3A) and in the final model soil type did not explain any of the variation in the model (Fig. 5D). However, regarding rooting ability, soil type explained 11.4 % of the variation (Fig. 5E). Cuttings in the acidic and organic soil (B) were less likely to develop larger roots (*b* = −0.49, s.e. = 0.23) than cuttings in the alkaline and mineral-rich soil (A) (threshold intercept 3|4: 2.63, s.e. = 0.40), and thus acidic and organic soil (B) had a negative effect on rooting ability (Fig. 6E).

The effect of site characteristics (i.e. habitat and plot number) differed between leaf production and rooting ability (Figs 5 and 6). Both leaf production and rooting ability were influenced by plot when included as a random factor, whereas rooting ability was additionally affected by different habitat types, which explained 13 % of the variation (Fig. 5E). Cuttings from slopes (*b* = 0.64, s.e. = 0.49) and riverbeds (*b* = 0.43, s.e. = 0.52) showed better rooting ability, compared to cuttings from ridges (threshold intercept 3|4: 2.63, s.e. = 0.40) (Fig. 6E). Notably, the distribution of cytotypes across habitats was uneven, with most cuttings from slopes and riverbeds being polyploids, and most cuttings from ridges being diploid (Supplementary Data Table S8, Appendix S3).

Site characteristics (i.e. habitat type and transect) also varied between capsule production and seed germination (Figs 5 and 6). Seeds from continental valleys (Endalen, Todalen, Bolterdalen and Foxdalen) were more likely to germinate (*b* = 2.56–3.20, s.e. = 0.19–1.26) than seeds from the most coastal valley, Bjørndalen (intercept: −4.05, s.e. = 1.21) (Fig. 6C), and seeds from ridges (intercept: −4.05, s.e. = 1.21) were more likely to germinate than seeds from riverbeds (*b* = −1.01, s.e. = 0.37) (Fig. 6C).

Generally, for all the reproductive outcomes, the AIC weights (*w*_i_) differed only marginally among the top five models, indicating little difference in the support for including phenotypic variation and site characteristics (Supplementary Data Tables S2–S6, Appendix S2). Consequently, there is some uncertainty related to the model selection and whether growth form should be included in models for capsule production (Table S2, Appendix S2) and seed germination (Table S4, Appendix S2), or whether habitat, as a fixed effect, should be included in models for seed production (Table S3, Appendix S2), leaf production (Table S5, Appendix S2) and rooting ability (Table S6, Appendix S2).

## DISCUSSION

This study investigated how cytotype, phenotypic variation, site characteristics or other covariates affect reproductive strategy in *S. oppositifolia*. *Saxifraga oppositifolia* is recognized for its significant phenotypic and genetic variability. Former studies from, for example, the Canadian Arctic and Svalbard have associated the different growth forms and reproductive differences with ecotype variation (Teeri, 1973; Crawford and Abbott, 1994; Crawford *et al*., 1995; Kume *et al*., 1999; Opała-Owczarek *et al*., 2018). Recent investigations have assigned some of the observed variation (e.g. growth form, habitat heterogeneity) to autopolyploidy (Eidesen *et al*., 2013, 2025). Yet, so far, no ecological niches have been exclusively associated with polyploids. Through this study, we found that autopolyploidy influenced two of the three investigated outcomes on sexual reproduction (i.e. capsule and seed production), and both parameters of vegetative investment (i.e. leaf production and rooting ability). Our findings support a shift in reproductive strategy towards higher vegetative investment and lower investment in sexual reproductive outcome in polyploids than in diploids. This shift towards vegetative reproduction in polyploids may enable a differentiation in their ecological niche optimum. It serves as the missing link in how polyploids may establish and compete with diploids within mixed-cytotype populations. The connection between cytotypes and reproductive strategy finally bridges the long-existing gap in understanding the evolutionary mechanisms behind phenotypic variation in *S. oppositifolia*, at least in Svalbard.

### Different reproductive modes and ecological differentiation

Our results showed that diploid *S. oppositifolia* in Svalbard invests primarily in sexual reproduction, with, for example, considerably higher seed set than polyploids (Figs 4 and 6). Good seed set in *S*. *oppositifolia* depends on sufficient insect visitation (Stenström and Molau, 1992; Gugerli, 1998), and maximum seed set in *S. oppositifolia* occurs when both Diptera and *Bombus* (Hymenoptera) are present in the pollinator guild (Kevan, 1972). However, pollen limitation has been reported from both alpine and subarctic environments (Stenström and Molau, 1992; Gugerli, 1998), and only Diptera occur in Svalbard (Coulson *et al*., 2024), implying that *S. oppositifolia* is probably strongly pollen-limited in Svalbard due to the lack of efficient pollinators. This raises the question of how diploid *S. oppositifolia* manages to sustain sufficient seed production under such conditions. Early-flowering species tend to exhibit higher outcrossing rates and are typically associated with habitats that melt out early, such as ridges, which provide a relatively long growing season (Molau, 1993). In Svalbard, *S. oppositifolia* is among the earliest arctic–alpine plants to flower, and diploid cytotypes fully dominate in habitats that melt out early (Eidesen *et al*., 2025). These habitats allow an early onset of flowering and increase the probability of successful pollination, because competition for the few available pollinators is still low compared to later in the growing season (Kevan, 1972; Molau, 1993; Kehrberger and Holzschuh, 2019). Consistent with this, pollinator visits on *S. oppositifolia* have only been recorded early in the season (Gillespie and Cooper, 2022). Furthermore, a longer growing season increases the probability that seeds will ripen. Thus, diploid *S. oppositifolia* in Svalbard fits the characteristics of a ‘pollen risker’: an early-flowering, predominantly outcrossing species that faces a high risk of pollen limitation due to sparse pollinator activity early in the season, but secures some seed output (Molau, 1993).

Diploids also occur in lower frequencies in neighbouring habitats that melt out later, where they co-occur with polyploids. Pollen limitation and time for seed-ripening in these habitats are more severe than in ridges and can affect local seed recruitment. Surprisingly, there was no difference in seed set between habitats (Fig. 6B). Yet, germination was less successful in seeds from habitats with later snowmelt and shorter growing seasons (e.g. slopes and riverbeds, Fig. 6C), which might affect local recruitment of diploids. However, *S. oppositifolia* has efficient seed dispersal, and seed rain from ridge habitats probably secures recruitment into adjacent areas.

Compared to diploids, polyploids showed lower investment in sexual reproduction (Fig. 4). Polyploids produced fewer capsules and seeds per capsule than diploids (Fig. 4A, B), in line with previous findings of flower and capsule production (Eidesen *et al*., 2025). On the other hand, tetraploids and triploids invested more than diploids in vegetative propagation, with higher leaf production (Fig. 3A), and more than five and three times, respectively, higher percentage of cuttings with well-developed and branching roots (rooting stage 4, Fig. 3B).

A shift in reproductive mode can be a strong mediator of ecological differentiation (Alonso-Marcos *et al*., 2019). Polyploids are predominantly distributed in less exposed habitats with later snowmelt and shorter growing seasons (e.g. slopes and riverbeds, Supplementary Data Table S8, Appendix S3), and experience even stronger pollinator limitations than early-flowering diploids. Short growing season with limited light conditions, and strong pollinator limitation may facilitate reproductive assurance strategies such as asexual reproduction and/or selection towards selfing or cleistogamy in polyploids (Yang and Kim, 2016). There is currently no evidence suggesting that the different cytotypes in *S. oppositifolia* differ in selfing ability. *Saxifraga oppositifolia* has strongly protogynous flowers, limiting selfing (Stenström and Molau, 1992), and seed set from selfing has been shown to be low (Kevan, 1972; Stenström and Molau, 1992; Gugerli, 1998). However, it should be noted that none of the former crossing or selfing experiments in *S. oppositifolia* (Kevan, 1972; Stenström and Molau, 1992; Gugerli, 1997, 1998) have accounted for putative cytotype variation. We therefore stress the need to conduct new crossing or selfing experiments between and within different cytotypes.

Vegetative propagation is generally more cost-efficient than asexual seed production (Lei, 2010). In habitats with shorter growing season and limited time for seed development, cuttings may effectively function as dispersal units. Enhanced vegetative propagation in triploids and tetraploids is therefore clearly advantageous and may partly explain the uneven distribution of cytotypes along the snow distribution gradient. Tetraploids have shown increased leaf mass area (LMA) and improved drought tolerance, which reduces the risk of drying out for newly fragmented cuttings (Eidesen *et al*., 2025). While seeds are better adapted for long-distance dispersal, *S. oppositifolia* cuttings are efficient for short-distance establishment in habitats with a short growing season and probably suffice for polyploid recruitment within mixed cytotype populations.

Faster growth may also be particularly beneficial when competing for ground space. Kume *et al*. (1999) found that the trailing growth form of *S. oppositifolia* had higher rooting ability and expanded faster than cushions in habitats disturbed by flooding. As the population density of trailing plants increased with successional changes, they further suggested that trailing plants had higher tolerance for competition (Kume *et al*., 1999). More effective vegetative investment is probably advantageous in habitats with stronger competition for light from neighbouring plants. Experimental warming studies from the International Tundra Experiment (ITEX) indicate that *S. oppositifolia* shows little sensitivity to increased warming, but it is relatively intolerant of shading by rapidly growing neighbouring species (Stenström *et al*., 1997). Several other examples of niche differentiation in autopolyploids exist (e.g. McIntyre, 2012; Thompson *et al*., 2014; Sonnleitner *et al*., 2016; Visger *et al*., 2016), confirming the common expectation that polyploids evolve broader ecological tolerance than their diploid progenitors (Spoelhof *et al*., 2017), but only in a few cases has this been coupled with a shift in reproductive mode, as we see in *S. oppositifolia*. For instance, in the grass *Paspalum intermedium* (Karunarathne *et al*., 2018), introduction of apomixis is associated with an increased ecological tolerance of polyploids, promoting a shift to habitats inaccessible to diploids.

### Fewer but equally good seeds?

Although polyploids invest less in sexual reproduction and produce fewer capsules and seeds, the germination percentage did not differ between diploid and tetraploid seeds (Table 4, analysis C), suggesting that these later-generation tetraploids are neither reproductively challenged in terms of postpollination prezygotic nor postzygotic intrinsic reproductive isolation barriers. Germination percentage among different cytotypes may differ in a natural environment. A study on various Arctic plant species, including *S. oppositifolia*, found that seed germination rates were higher under optimal conditions in a climate chamber compared to outdoor germination with natural conditions (Müller *et al*., 2011). Without addressing putative differences related to cytotype, the germination rates were >60 % in *S. oppositifolia* under optimized conditions indoors, whereas the germination percentage *in situ* was just 2 % in the first year, but 20 % after 2 years (Müller *et al*., 2011). Consequently, the seed germination percentage observed in the present study might have been overestimated relative to germination under natural conditions.

The obtained germination percentage varied from 0 to 56 % (Fig. 4C), i.e. much lower than what was previously reported under controlled conditions (Müller *et al*., 2011). In contrast to our study, the seeds used in the experiment by Müller *et al*. (2011) were stratified under natural conditions, being stored outside over winter before the germination experiment started. Thus, stratification conditions may have influenced our germination percentages, and seeds of different cytotypes may require different stratification conditions to break dormancy. Physiological seed dormancy has, for instance, been found to increase with elevation in the Pyrenean *Saxifraga longifolia* (Cotado *et al*., 2020). The fact that diploids are dominating in habitats with more extreme temperature ranges, both summer and winter (Eidesen *et al*., 2025), may suggest that diploid seeds display different levels of seed dormancy than tetraploids.

Triploid *S. oppositifolia* plants had a seed production close to zero (Fig. 4B) and were not represented in the germination experiment. Accordingly, they seem to rely on vegetative propagation only. Triploids are usually reproductively challenged through chromosomal pairing problems because of their odd number of chromosomes (Ramsey and Schemske, 1998). Whether the triploids are ephemeral hybrids recurrently produced between tetraploids and diploids and represent a triploid bridge as part of ongoing autopolyploidization (Ramsey and Schemske, 1998), or constitute an established lineage relying on vegetative propagation, is currently unknown.

### Polyploid establishment and survival in the long run

Our findings strengthen the expectation that polyploids are more often associated with vegetative reproduction (Gustafsson, 1948; Osterman *et al*., 2025). The importance of this association for polyploid establishment has been emphasized by stochastic and simulation modelling (Spoelhof *et al*., 2020; Van Drunen and Friedman, 2022), and phylogenetic comparative analyses have confirmed a positive association between polyploidy and vegetative reproduction among flowering plants (Herben *et al*., 2017; Van Drunen and Husband, 2019). Nevertheless, the relatively few empirical studies on smaller scales have provided mixed results. Some studies report that polyploids invest more in vegetative growth than their diploid relatives, as found for instance in *Solidago gigantea* (Schlaepfer *et al*., 2010), *Vaccinium vitis-idea* (Wakui and Kudo, 2021) and *Pilosella rhodopea* (Šingliarová *et al*., 2023). Van Drunen and Husband (2018*a*) found the same pattern for neopolyploids of *Chamerion angustifolium*, whereas Baldwin and Husband (2013) found that established polyploids of the same species were less clonal than diploids. Another study reported neopolyploids of *Fragaria vesca* to be less clonal than diploids (Van Drunen and Husband, 2018*b*), and Keeler (2004) found no difference in clonality between cytotypes of the grass *Andropogen geradii*. Such opposing results probably underscore the varying nature of vegetative reproductive modes and functions in plants (Klimeš *et al*., 1997), with the few species mentioned above representing several different strategies, ranging from creeping stems and short persistent rhizomes, to expanding stolons and independent plantlet development. In addition, the association between vegetative production and polyploidy seems to vary among the major angiosperm clades, as suggested by Van Drunen and Husband (2019). Based on a comparative phylogenetic study including 1751 species, they found, for instance, a higher association between clonal growth and polyploidy in the Asteraceae and Rosid clades than in the Monocot clade. It is also likely that the association between polyploidy and vegetative reproduction varies across habitats and geographical regions, as vegetative reproduction is unevenly distributed over environments and habitats (Klimeš *et al*., 1997) and the frequency of polyploidy is strikingly uneven on a global scale (Rice *et al*., 2019). More small-scale ecological and evolutionary studies are probably needed to fully understand the evolutionary interplay of the association between polyploidy and vegetative reproduction.

Polyploidization may trigger the development of vegetative reproductive modes that were not present in the diploid progenitors (Otto and Whitton, 2000). The change in reproductive mode following polyploidization is generally discussed as an advantage for successful establishment and reduced risk of immediate backcrossing with their diploid progenitor (Otto and Whitton, 2000; Ramsey and Schemske, 2002). However, genome doubling may also just enhance an already existing ability by increasing the number and subsequent differentiation of meristems (Müntzing, 1936; Husband *et al*., 2013). Since *S. oppositifolia* diploids are capable of vegetative propagation (Fig. 3B), this reproductive mode has probably been enhanced in triploids and tetraploids, rather than originating from a polyploidization event. Eidesen *et al*. (2025) found trailing diploids to be signiﬁcantly more frequent in dry, exposed ridges than in riverbeds, suggesting that trailing growth probably plays different adaptive roles in diploids and polyploids (Eidesen *et al*., 2025). Thus, the enhancement of vegetative growth in *S. oppositifolia* polyploids has probably produced a novel selective trait associated with trailing growth. A shift in resource allocation from sexual reproduction to vegetative propagation in polyploids can limit mixing with diploids and potentially ‘a triploid block’ (Ramsey and Schemske, 1998; Husband and Sabara, 2004). By reducing sexuality, polyploids may mitigate intercytotype competition in a mixed-cytotype population dominated by diploids.

However, whether the observed shift towards a vegetative reproductive mode in polyploids in *S. oppositifolia* in Svalbard is a direct consequence of the polyploidization event itself or instead evolved more gradually over time cannot be concluded based on current knowledge. Müller *et al*. (2012) proposed that the origin of the tetraploids pre-dates the colonization of Svalbard, which suggests that Svalbard may represent a zone of secondary contact. Refugial separation of diploid and tetraploid lineages prior to colonization of Svalbard could then have promoted divergence in reproductive mode between cytotypes after the establishment of a tetraploid lineage. Whether each cytotype colonized Svalbard from separate source regions, or from one or several source regions with mixed populations, is currently unknown. Both scenarios are plausible, as very strong phylogeographical connectivity based on nuclear markers has been found between Greenland and Svalbard (Winkler *et al*., 2012), and all cytotypes recorded in Svalbard have also been reported from Greenland (Eidesen *et al*., 2013, 2025; Elven *et al*., 2023; Gago and Clemente, 2023). More extensive mapping of cytotypes, combined with phylogeographical analyses, is needed to obtain a fuller picture of the cytotype history.

In general, vegetative propagation reduces genetic diversity and may be a potential challenge for the long-term survival of tetraploid *S. oppositifolia*. However, Müller *et al*. (2012) found no reduced genetic variation in tetraploid *S. oppositifolia* plants compared to diploids based on amplified fragment length polymorphisms (ALFPs). A study on genetic diversity at different spatial scales in *S. cernua*, which mainly reproduces clonally via bulbils, found that even occasional sexual reproduction in exceptionally good years can maintain the genetic diversity in an Arctic clonal plant (Gabrielsen and Brochmann, 1998). Since the limited number of seeds produced by tetraploids in our study germinated as effectively as diploid seeds, it may be sufficient to preserve the genetic diversity within the polyploid population, while increased vegetative propagation simultaneously limits diploid mixing. A recent study on the autopolyploid complex of *Campanula rotundifolia* revealed evidence for low reproduction between diploid and tetraploid cytotypes and that cytotype-mixing occurred primarily via unreduced gamete production (Sutherland and Galloway, 2021). Previous studies on the production of unreduced gametes in *S. oppositifolia* found no significant differences among cytotypes and that the overall levels of unreduced gamete production were not higher for *S. oppositifolia* than commonly found in other angiosperm species (Eidesen *et al*., 2025). As a result, *S. oppositifolia* polyploids may not depend on a physical barrier or spatial segregation to prevent cytotype interbreeding and the risk of becoming extinct in a few generations due to a large portion of ineffective pollination events, as predicted by the minority cytotype exclusion (MCE) principle (Levin, 1975; Husband, 2000).

### Confounding effects

In this study, we ensured an adequate representation of the various habitat types where *S. oppositifolia* is growing; however, the distribution of cytotypes within these habitats was uneven (Supplementary Data Table S8, Appendix S3). Consequently, most cuttings collected from ridges were diploid, whereas cuttings from slopes and riverbeds were predominantly polyploids. This disparity suggests that the influence of autopolyploidy may confound the habitat effect. While cytotypes significantly influenced vegetative propagation, the impact of different habitat types appears less pronounced than previously assumed when accounting for cytotype. Moreover, diploids with the cushion growth form and short internodes were not represented in the rooting experiment as they could not be collected without damaging the ramets. The enhanced rooting ability has primarily been linked to the trailing growth form (Hagen, 2002), suggesting that the inclusion of diploid cushions in the rooting experiment would have negatively impacted the result obtained for diploids and probably led to an even greater difference in rooting ability between the cytotypes (Fig. 3B).

### The evolutionary importance of autopolyploidy

Average global temperature is increasing, particularly in alpine regions at high latitudes (Pepin *et al*., 2022), resulting in significant and rapid habitat changes in the arctic–alpine archipelago of Svalbard (Adakudlu *et al*., 2019; Karlsen *et al*., 2024). With a warmer climate and increased competition from migrating southern species (Pykälä, 2017), Arctic plant species may become evolutionary losers (Somero, 2010). Range-restricted, arctic–alpine species have already shown severe contractions and have been some of the first species to go extinct due to recent climatic changes (Watts *et al*., 2022). With these changing conditions, species may need rapid evolutionary adaptations to either cope with or evade new habitats. Polyploidization, demonstrated in this study as an evolutionary mechanism for niche shift, may contribute by altering already present abilities or introducing new favourable traits and reproductive modes to better withstand the changing Arctic environment.

## CONCLUSION

Our findings confirm a shift in reproductive strategy characterized by reduced sexual investment and increased vegetative investment in polyploid individuals of *S. oppositifolia* in Svalbard. This shift may enhance the survival of polyploids in habitats with shorter growing seasons and limited pollinator availability, thus explaining their higher frequencies in such environments. Autopolyploidy is indeed an evolutionary mechanism that may enable Arctic species to adapt to the ongoing rapid environmental changes.

## Supplementary Material

mcag116_Supplementary_Data

## Data Availability

The regression search algorithms used here can be found at GitHub: https://github.com/trondreitan/regress_search.git. Code for statistical analyses: https://github.com/IVTjessem/should-i-sex-or-should-i-grow.git.

## SUPPLEMENTARY DATA

Supplementary data are available at Annals of Botany online and consist of the following. Appendix S1: Description of the variables used in the statistical analyses. Appendix S2: The five best models from AICc model comparison procedures, AIC weights and final best models of all reproductive outcomes. Appendix S3: Distribution of cuttings across habitat types (ridge, slope and riverbed) and cytotypes (2*x*, 3*x* and 4*x*).

## AI ASSISTANCE

AI was used as an aide in the writing of the manuscript: for English improvement and rephrasing, but not for writing original text.
