# Supplementary material for: A shift in species niche through autopolyploidy: from sexual to vegetative reproduction in Saxifraga oppositifolia
Source: Ann Bot. 2026 Apr 29;138(2):651–66. doi: 10.1093/aob/mcag116 (PMC13454688; doi:10.1093/aob/mcag116)
Supplement: Supplementary file 1 [file mcag116_supplementary_data.pdf]

Appendix S1: Description of the variables used in the statistical analyses

Supplementary Table 1. Description of the relevant covariates, which presumably could explain variation in the five reproductive outcomes of *Saxifraga oppositifolia* (Table 2). ID corresponds to a per-sample random effect.

**Covariates (variable type)**	**Description**
No. of flowers (integer)	0–208 flowers (total number of closed, open, and withered) per plant. Flowers were recorded twice (June and July) during the field season of 2020. For a more thorough description, see Eidesen *et al*. (2025).
Growth form (integer)	0–100% cushion. Recorded and described in Eidesen *et al.* (2025).
Habitat (nominal)	3 categories: riverbed, slope, ridge. Recorded and described in Eidesen *et al.* (2025).
ID (character)	Individual identification of each *Saxifraga oppositifolia* plant.
No. of seeds (numerical)	0–338 seeds.
Plot (nominal)	15 categories: 2, 3, 4, 5, 6, 7, 8, 9, 10, 11, 12, 13, 14, 15, 16. Established and described in Eidesen *et al.* (2025).
Seed weight (numerical)	0.00001–0.0057 gram.
Soil type (nominal)	2 categories: mineral soil (A), organic soil (B).
Transect (nominal)	5 categories: Bjørndalen, Bolterdalen, Endalen, Foxdalen, Todalen. Established and described in Eidesen *et al.* (2025).

Appendix S2: Five best models from AICc model comparison procedures, AIC weights, and final best models of all reproductive outcomes

AIC weights (w_i) can be interpreted as conditional probabilities for models and make it easier to interpretate the results of an AIC model comparison procedure (Wagenmakers & Farrell, 2004). The AIC weights were used to evaluate the evidence for each of the five best models and to compare them thereafter. The weights were calculated from AIC values by the following formulae:

$$w_i = \frac{e^{-0.5AIC_i}}{\sum_{j=1}^{\#models} e^{-0.5AIC_j}} = \frac{e^{-0.5(AIC_i - AIC_{best})}}{\sum_{j=1}^{\#models} e^{-0.5(AIC_j - AIC_{best})}} = \frac{e^{-\Delta AIC_i/2}}{\sum_{j=1}^{\#models} e^{-\Delta AIC_j/2}}$$

Analysis A – Capsule production

Supplementary Table 2. The five best models obtained from the explorative analysis used to test whether capsule production in *Saxifraga oppositifolia* was affected by covariates, carried out by the function *regress.ic.search*, which stepwise added, removed, or replaced single or interacting (:) covariates, including both fixed and random (|) effects. Second-order Akaike Information Criterion (AICc) and AIC-weight (wi) were used to evaluate and compare models.

**Model**	*Habitat*	*Transect*	*Growth form*	(1|*Plot*)	(1|*ID*)	*Growth form*: *Habitat*	*Growth form*: *Transect*	**AICc**	w_i
**1**	x				x	x	x	2886.36	0.27
**2**	x		x		x	x	x	2886.52	0.25
**3**	x	x			x	x		2886.98	0.20
**4**	x	x			x	x	x	2887.64	0.14
**5**	x			x	x	x	x	2887.67	0.14

Analysis B – Seed production

Supplementary Table 3. The five best models obtained from the explorative analysis used to test whether seed production in *Saxifraga oppositifolia* was affected by covariates, carried out by the function *regress.ic.search*, which stepwise added, removed, or replaced single or interacting (:) covariates, including both fixed and random (|) effects. Second-order Akaike Information Criterion (AICc) and AIC-weight (wi) were used to evaluate and compare models.

**Model**	*Habitat*	*Growth form*	(1|*ID*)	(*Growth form* | *Plot*)	*Habitat*: *Growth form*	**AICc**	w_i
**1**			x	x		1350.28	0.28
**2**	x		x			1350.70	0.22
**3**	x	x	x		x	1350.75	0.22
**4**			x			1351.35	0.16
**5**		x	x	x		1351.96	0.12

Analysis C – Seed germination

Supplementary Table 4. The five best models obtained from the explorative analysis used to test whether seed germination in *Saxifraga oppositifolia* was affected by covariates, carried out by the function *regress.ic.search*, which stepwise added, removed or replaced single or interacting (:) covariates, including both fixed and random (|) effects. Second-order Akaike Information Criterion (AICc) and AIC-weight (wi) were used to evaluate and compare models.

**Model**	*Habitat*	*Transect*	*Growth form*	*Seed weight*	(1|*ID*)	*Habitat: Seed weight*	**AICc**	w_i
**1**	x	x	x		x		280.91	0.34
**2**	x	x			x		282.06	0.19
**3**	x		x		x		282.07	0.19
**4**	x				x		282.35	0.16
**5**	x			x	x	x	282.98	0.12

Analysis D – Leaf production

Supplementary Table 5. The five best models obtained from the explorative analysis used to test whether leaf production in *Saxifraga oppositifolia* was affected by covariates, carried out by the function *regress.ic.dredge*, which looked through all possible combinations for potential single or interacting (:) covariates, including both fixed and random (|) effects. Second-order Akaike Information Criterion (AICc) and AIC-weight (wi) were used to evaluate and compare models.

**Model**	*Habitat*	*Soil*	(1 |*Plot*)	(*Soil* | *Plot*)	**AICc**	w_i
**1**			x		249.13	0.45
**2**	x		x		250.40	0.24
**3**		x	x		251.18	0.16
**4**	x	x	x		252.47	0.09
**5**				x	253.23	0.06

Analysis E – Rooting ability

Supplementary Table 6. The five best models obtained from the explorative analysis used to test whether rooting ability in *Saxifraga oppositifolia* was affected by covariates, carried out by the function *regress.ic.dredge*, which looked through all possible combinations for potential single or interacting (:) covariates, including both fixed and random (|) effects. Second-order Akaike Information Criterion (AICc) and AIC-weight (wi) were used to evaluate and compare models.

**Model**	*Habitat*	*Soil*	(1|*Plot*)	*Habitat:Soil*	**AICc**	w_i
**1**	x	x	x		710.99	0.39
**2**		x	x		711.50	0.30
**3**	x		x		713.09	0.14
**4**			x		713.51	0.11
**5**	x	x	x	x	714.56	0.07

Supplementary Table 7. The final models that best explain variation in reproductive outcomes (A-E) in *Saxifraga oppositifolia.* The models include potential single or interacting (:) covariates, with fixed and random (|) effects. ID corresponds to a per-sample random effect.

**Analysis**		**Final model**
**A**	Capsule production (sexual reproduction)	~ Cytotype + Habitat + (1|ID) + Growth.form:Transect + Growth.form:Habitat
**B**	Seed production (sexual reproduction)	~ Cytotype + (1|ID) + (Growth.form|Plot)
**C**	Seed germination (sexual reproduction)	~ Habitat + Transect + Growth.form + (1|ID)
**D**	Leaf production (vegetative propagation)	~ Cytotype + (1|Plot)
**E**	Rooting ability (vegetative propagation)	~ Cytotype + Habitat + Soil + (1|Plot)

Appendix S3: Distribution of cuttings across habitat types (ridge, slope and riverbed) and cytotypes (2x, 3x and 4x)

Supplementary Table 8. Number of the surviving *Saxifraga oppositifolia* cuttings across habitat types (ridge, slope and riverbed) and cytotypes (2x, 3x and 4x), used in the rooting ability and leaf production experiment.

Habitat	2x	3x	4x	Total
**Ridge**	71	15	4	**90**
**Slope**	14	43	25	**82**
**Riverbed**	6	25	62	**93**
**Total**	**91**	**83**	**91**	**-**
